# Reusable Embedded Microcoils for Magnetic Nano-Beads Trapping in Microfluidics: Magnetic Simulation and Experiments

**DOI:** 10.3390/mi11030257

**Published:** 2020-02-28

**Authors:** Olivier Lefebvre, Hong Ha Cao, Meritxell Cortés Francisco, Marion Woytasik, Elisabeth Dufour-Gergam, Mehdi Ammar, Emile Martincic

**Affiliations:** 1 Université Paris-Saclay, CNRS, Centre de Nanosciences et de Nanotechnologies, 91120 Palaiseau, France; meritxell.cortes83@gmail.com (M.C.F.); marion.woytasik@c2n.upsaclay.fr (M.W.); elisabeth.dufour-gergam@c2n.upsaclay.fr (E.D.-G.); mehdi.ammar@c2n.upsaclay.fr (M.A.); 2 School of Chemical Engineering, Hanoi University of Science and Technology, Hanoi 10000, Vietnam; caohonghabk@gmail.com (H.H.C.); emile.martincic@c2n.upsaclay.fr (E.M.)

**Keywords:** planar microcoils, microfluidics, polydimethylsiloxane (PDMS), magnetic beads trapping, magnetic field simulation

## Abstract

In this study, a microfluidic chip with integrated coil was designed and fabricated for the aim of effectively trapping magnetic nanobeads (Adembeads^®^, 300 nm) and measuring the chip’s temperature during the working time. In addition, a reversible technique of bonding Polydimethylsiloxane (PDMS) channels was presented. This bonding process used a coating layer of CYTOP^®^product as a protection, insulation and low-adhesion layer. The reversible packaging technique allows the bottom substrate to be reused, possibly equipped with sensors, and to use a disposable microchannels network. The FE method was employed to calculate the magnetic field and power consumption by the ANSYS^®^ version 12.1 software. Merit factors were defined in order to synthetically represent the ability of the simulated coil to trap beads for a unit power consumption, i.e. a given heat generation. The simulation results propose a new approach to optimize the design criteria in fabricating planar microcoils. The optimal microcoils were fabricated and then used to realize a magnetic immunoassay in a microfluidic chip. The aim was to integrate these microcoils into a lab-on-chip and obtain a fast and highly sensitive biological element detection.

## 1. Introduction

Lab-on-a-chip devices have changed the way in which biological, medical and chemical analysis processes are being conducted by miniaturizing and automating systems. They are able to perform multiple processing steps of biology and chemistry onto tiny chips [1,2,3]. Bio-chips are designed to integrate a wide variety of functions such as valves, pumps, heat controller, magnetic generators, etc., which are commonly required in biological process [4,5]. Among different ways to control beads (such as microswimmer, micro-pump, microactuator [6,7,8,9,10,11,12,13]) this paper focuses on magnetic beads trapping with microcoils in microchannels on an integrated microfluidic chip.

Manipulating magnetic micro/nano beads in microfluidic chips can be done efficiently with permanent/electro magnets [4,14,15]. The permanent magnet is known to make a strong magnetic flux density, i.e., from few hundreds mT to 1 T [16,17,18,19], thus generating a strong attraction force. However, the permanent magnets only generate a permanent field. Consequently, generating or diminishing the magnetic field requires mechanically moving the magnet. Therefore, the magnetic field cannot be turned off immediately but within a certain response time. 

Electromagnets can realize the same attraction function as permanent magnets. Furthermore, electromagnets can generate a controlled magnetic field and can be rapidly switched. Despite this advantage, the magnetic field generated by electromagnets of reduced dimensions (micro-electromagnets) is much lower than permanent’s magnets. The base elements of electromagnets are coils. When miniaturizing below 1 mm dimension, planar coils are usually fabricated with a planar shape. The magnetic flux density ranges then from some mT to hundreds of mT [20,21]. A high current requiring extra cooling or designs with extra parts is required to raise the magnetic field intensity. For planar coils, it can be improved with either a magnetic material sublayer or yoke. Some examples of 3D micromachined coils using magnetic material that could be used for trapping with an addition of a microchannel were also published [22,23,24]. 

Joule effect heating is a major concern when using micro-electromagnets [5,25,26]. Especially for bio-application, while trapping and “physical” functions used in microfluidic chips can be operated up to the water boiling temperature, handling bio-species in the microfluidic chip requires that the temperature maintained below a much lower temperature in order to keep the binding reactions between species possible or to prevent their destruction. The microfluidic chip heating issues have been dealt with in different manners: in [25], an extra set of microfluidic channels have been fabricated in order to convey a cooling fluid; in [27], the microfluidic chip is operated over a Peltier thermoelectric module to regulate the chip’s temperature below 37 °C. 

Despite these thermal issues, the thermal design of microcoils is scarcely studied. Limiting the coil’s thermal generation by design could be a way of reducing the cooling efforts, or even make extra cooling unnecessary. In [5], trapping current control strategies are implemented to prevent the chip from heating up to an undesirable temperature. 

We propose in this paper a new approach in designing coils for trapping nano-magnetic particles with the objective of maximizing the trapping efficiency to heat generation ratio. The operating temperature range of 30 °C to <40 °C is a biocompatible temperature range, suitable for the bio-assay (on-chip ELISA) to be further implemented. Ansys^®^ version 12.1 Finite Element Modelling (FEM) software was used in this work. FEM was used to predict the power/heat generation without manufacturing a whole set of microcoils [2,21,28]. Simulated values can then be obtained even for designs that would suppose a low fabrication yield. 

In the second part, in order to validate simulations, microchips with integrated coils were fabricated. In Polydimethylsiloxane (PDMS) based microfluidic chips, strong “PDMS to PDMS” bonding could be easily obtained by plasma treatments combining with thermal treatments [29]. However, the bonding is irreversible and consequently the chips are disposable. In our case, reusing the microcoil embedded on the bottom substrate is feasible. In this study, an advanced chip packaging technique was developed for the advantage of reusing the bottom substrates by using the conformal coating layers as a protection/insulation layer and an anti-adhesive layer on the surface of coils: CYTOP^®^, a Teflon-like perfluoropolymer, was previously used in [30].

## 2. Optimal Coils Design

### 2.1. Magnetic Field Generation

The Biot–Savart law defines the magnetic field **H** generated at some point in space by a current *I*, passing in a wire of finite length d***ℓ***:

(1) $$dH=\frac{I}{4\pi }\frac{dl\times r}{r^{2}}$$

where **r** is the distance from that point to the finite straight wire, **r** is the displacement vector from the element d***ℓ*** to the point.

The relationship between the magnetic field **H** and the magnetic flux density **B** is given by:

**B** = μ_0_**H** (1 + χ_m_) = μ_0_ μ_r_**H** (2)

where χ_m_ is the magnetic susceptibility of material, μ_0_ is the permeability of free space: μ_0_ = 4π × 10^−7^ T·m/A, μ_r_ is the relative permeability of the material.

When a magnetic micro/nano bead is placed into a magnetic field, a magnetic force is exerted onto it. The magnetic force can be expressed by [31]:

(3) $$F_{m}=\frac{1}{\mu_{0}}\nabla \left(m.B\right)\approx \frac{1}{\mu_{0}}\left(m.\nabla \right)B$$

where *m* is the magnetic moment of the bead.

In the case of magnetic beads in a non-magnetic medium, the magnetic moment can be written: **m =** *V* μ_0_ **M =** *V* μ_0_ χ **H**, where **M** is the magnetization of bead, *V* is the volume of magnetic bead, χ is the susceptibility of the magnetic component of the bead. This relation explicitly means that the bead’s internal magnetic field is directly proportional to the external applied magnetic field. The Equations (2) and (3) can be re-written in the air:

(4) $$F_{m}=\frac{V\;\chi }{\mu_{0}}\left(B.\nabla \right)B$$

Equation (4) expresses that the exerted force is proportional to the product B modulus and its gradient. Then, it can be deduced that the trapping force is high when the gradient and magnitude of B (magnetic flux density) are high. A first obvious observation can be made when the B (magnetic flux density) is close to zero: the gradient is then also close to zero. A second observation is that even in the presence of a strong B, if the gradient is close to zero, almost no force is exerted on the bead. For convenience, it is much easier to represent either magnitude of B since FEM software primarily shows the B magnitude. Since all microcoils studied here have diameters ranging from some hundreds of μm to 1 mm, the *B*_max_/Coil Diameter ratio approximately represents the gradient of B. Indeed, having a strong B in a small space (such as the space occupied by a microcoil) means a strong gradient around the microcoil. Thus, for microcoils design considerations, a desired property has to be achieved: generating B as strong as possible. Finally, the B gradient can be high by changing its amplitude or direction. These important considerations were already pointed out in [31]. The next paragraph will refine these considerations on the same basis.

### 2.2. FE Simulation of Microcoils

**Figure 1:**
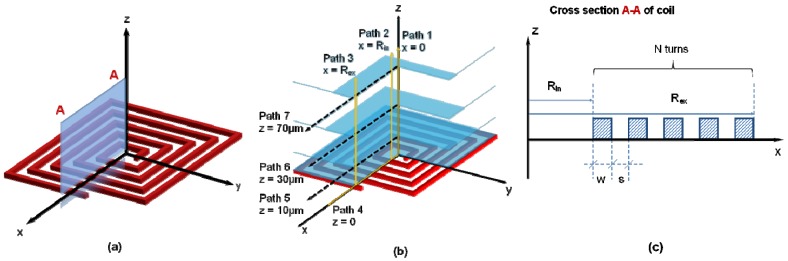
(**a**) The coil model showing the simulated cross section; (**b**). Positions of the magnetic magnitudes extracted from simulation results; (**c**). Parameters of coil model at cross section A-A; *w*, width of Cu wire; *s*, space between two Cu wires; *R_in_*, inner radius of coil; R*_ex_*, outer radius of coil.

FEM software (ANSYS^®^ version 12.1.) was used to calculate the magnetic field generated by microcoils. Then, 2D simulations were carried out, with the assumption that the length of each segment is infinite compared to the working distance. The study of the magnetic field can then be reduced to a longitudinal cut of the microcoil: half of the longitudinal cut is represented in Figure 1 [32]. The 2D simulations presented here implicitly contain the hypothesis that the region of interest, i.e., the microfluidic channel, is smaller than the radius of the microcoil and that the conductors are wound (i.e., not meander shape). 

The magnetic flux density is calculated for:

- Paths (1 to 3) along the *z*-axis at three different positions in *x*-axis: center (*x* = 0), inner wire (*x* = *R_in_*) and outer wire (*x* = R*_ex_*) of the coil.
- Paths (4 to 7) along the *x*-axis at different distances from the wire surface (Cu). *z* = 0; 10; 30; 70 μm.

Table 1 shows the parameters used for the simulations and the input values. The values were chosen based on the technical ability of manufacturing these magnetic coils in our cleanroom fabrication processes.

### 2.3. Simulation Results 

#### 2.3.1. Profile of the Magnetic Field

**Figure 2:**
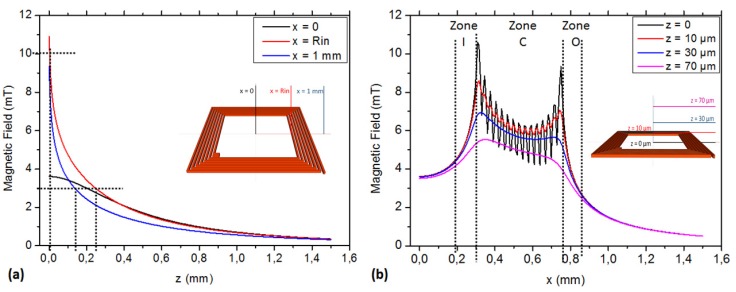
The profile of magnetic flux (B) of coils at path 1, 2, 3 (**a**) and 4, 5, 6, 7 (**b**). Coil: R_in_ = 300 μm; *w* = 10 μm; *s* = 10 μm; N = 25 turns, *h* = 15 μm.

Figure 2 shows the simulation results of the magnetic flux density on all paths (from path 1 to 7) above the surface of the square microcoil with the following parameters values: *R_in_* = 500 μm, *N* = 20 turns, *w* = 10 μm, *s* = 10 μm and *h* = 15 μm. 

The magnetic field intensity (B) modulus is higher in close vicinity to the coil. Equation (1) determination corresponds to the rough 1/x shape observed in Figure 2a. Conversely, the trapping capability is higher in the near vicinity (some μm above) of the coil’s surface. For microfluidic considerations, the trapping force depends on the distribution of magnetic force along *z*-axis [33]: it can be predicted to be high in the bottom of the microchannels but with a fast decrease. An order of magnitude can be deduced from Section 2.1 results: in z direction, the trapping force is approximately 10 times less at z = 0.2 mm compared to z = 0.

Figure 2b shows the B modulus in several paths parallel to the coil surface. As observed in previous paragraph, the B modulus decreases with higher distances to the coil’s surface. B modulus strongly decreases at the coil edges (*x* > 500 μm). The FEM original result is the strong B modulus ripple at a distance below *z* ≈ 10 μm (Zone C). According to Equation (4), B ripple and amplitude combine to improve the trapping efficiency of the coil in the near vicinity to its surface. The B modulus ripple is then a helping factor for trapping. Nevertheless, the B ripple cannot be considered as a primary trapping design parameter. In our case, the PDMS protecting layer is several μm thick (see Section 3.2.2). Only in few cases is the channel height below 10 μm, and then in the vast majority of cases, the B ripple will only help in a small fraction of the microchannel, just above the coil. The B modulus neglecting the ripple is the main parameter to be taken into account. 

Other coils were simulated varying the turn numbers (*N*), radius (R*_ex_*), width of copper wire (*w*), etc. The results showed a similar distribution of the magnetic field modulus, a similar decrease of B modulus, can be observed when the distance to the surface increases and a similar B ripple can be observed in the near surface vicinity.

Consequently, the microchannel should be rather wide and not very high, whenever the application allows such design. Furthermore, as the coil could not be directly in contact with the liquid flowing in the microchannel, the necessary protective layer must be fabricated as thin as possible. 

**Figure 3:**
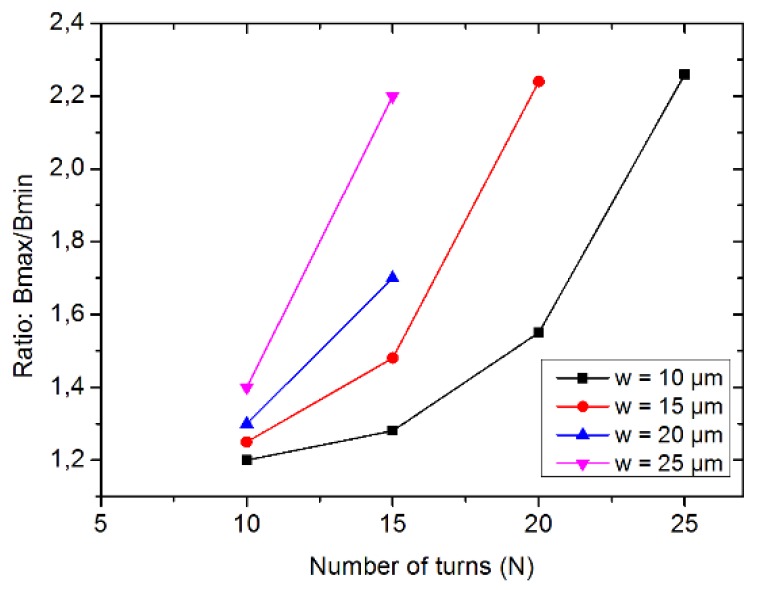
Homogeneity of the magnetic flux density: *B_max_/B_min_* for a given R*_ex_* value (1 mm).

Figure 2 suggests that B modulus is high over the surface of the coil and that the gradient is high in the edge’s vicinity (zones I and O). The trapping of the beads can then be stronger at the inner edge of the coil or at both edges. Neglecting the liquid flow considerations, B modulus flatness over the coil surface can account for the trapping homogeneity (the higher the unflatness, the higher the trapping homogeneity). *B*_max_/*B*_min_ ratio accounts for such homogeneity. The *B*_max_/*B*_min_ ratio for several coils of the same outer radius is represented in Figure 3. 

Figure 3 shows that homogeneously trapping is obtained for a low number of turns, which also means a low generated magnetic flux density and thus lower trapping capability.

#### 2.3.2. Coil Geometry Effects on Power Loss and Heating

Microcoils’ dimensions are limited by lithography resolution and deposition or plating technologies, which drive a significant minimum spacing between conductors. Then, the copper coils cannot use all the available space on the wafer surface. The surface filled by copper is represented by the filling ratio:

(5) $$r=\frac{N\;w}{R_{ex}}100\%$$

**Figure 4:**
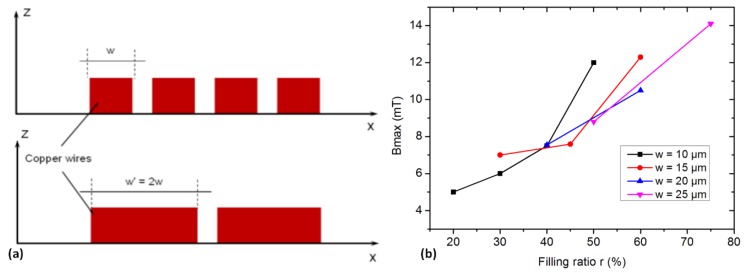
(**a**). Configuration for different widths of the conductors, (**b**). Effect of filling ratio on *B_max_* value.

We started with the spacings between conductors of *s* = 10 μm, which correspond to a safe lower limit when using the thick copper micro-molding process. For given spacings and widths of the conductors, the filling ratio increases with respect to *N* (Figure 4a). Its higher limit is given by the ratio *w*/(*s + w*): the width of the conductors to the sum of the width and spacing of the conductors. Figure 4b shows the maximum modulus of flux density *B_max_* (for R_*ex*_ = 500 μm) generated with coils for different conductors’ width and turn numbers (*w* and *N*) at path 5 (z = 10 μm). In this graph, the *B_max_* increases with the rise of *N* at a given *w* value. The obtained filling ratio values are between 20% and 75%. In our simulation, the current density was fixed with the expectation to determine the corresponding value of *B_max_*. From the obtained *B_max_*, the relationship between the number of turns and the width of the conductors was then determined. This relationship depends on the ratio of filling Cu (r) on the coil surface presented in Figure 4.

As the simulation input was chosen to be the current density, the product of the number of turns and current flowing through the coil (N.I.) is proportional to a given number of turns to the width of the conductors. It can be observed in Figure 4b that for a given coil configuration, the effective maximum flux density is roughly proportional to the filling factor. 

The maximum B modulus was obtained with the highest filling ratio (around 75%), which means that a design rule for trapping coils can be to make coils with conductors’ width larger than the spacing between conductors:

*w* >> *s* (6)

Figure 4b shows that the maximum B modulus is not always obtained for a wider conductor: the coil with *w* = 20 μm allows a *B_max_* of 10 mT but *w* = 15 μm allows a *B_max_* of 12 mT. When *w* is high, a space kept in the center of the coil cannot be filled with a conductor. As the limit of the filling ratio gets higher, the *B_max_* seems to not be as high as the results obtained from other configurations.

Despite the high interest for power efficiency to maximize the width of the wires, it can be noted that when conductors are very wide, the current concentrates in the inner section part of the conductor in order to take the lower length path from one terminal to the second one. This leads to higher power dissipation for wide conductors than for low width ones. The optimal number of turns is then given by a trade-off between the maximization of the filling factor while keeping the current density identical in the conductor’s section.

#### 2.3.3. Power Consumption and Merit Factors

The power loss by Joule effect in the coil is known to be

(7) $$P={R\;I}^{2}=V_{Cu}\;\rho_{Cu}\;J^{2}$$

where *I* is the current flowing through the coil, *J* is the current density, *ρ*_Cu_ is the resistivity of copper and *V*_Cu_ is the total volume of copper. 

**Figure 5:**
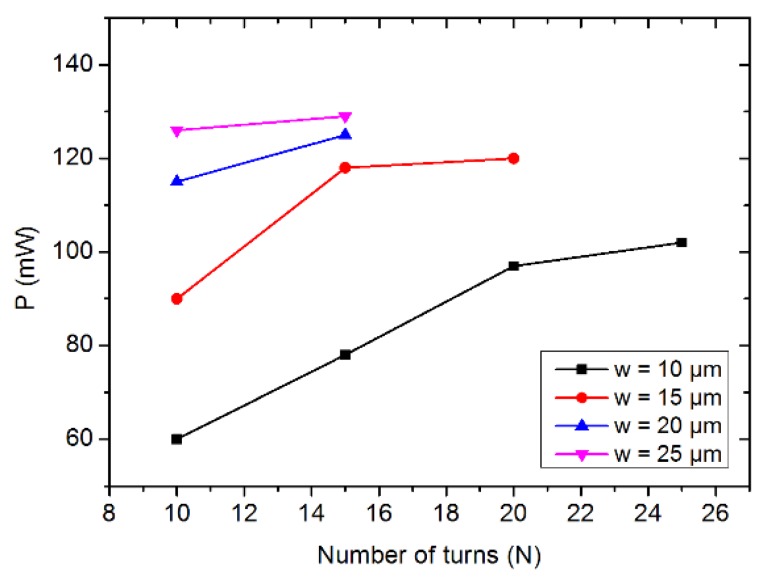
Power consumptions (P) in coil with different turns numbers and copper width (R*_ex_* = 500 μm).

Figure 5 shows the variation of power consumption in coils (R*_ex_* = 500 μm) as a function of the turn number for several widths of copper conductors.

Previous considerations drive to design coils with a high filling factor r: Bmax is maximized when filling ratio r is higher than 60%. The power consumption represented in Figure 5. shows that for given outer radius and width of the conductors, increasing the filling ratio (i.e., increasing *N*) only slightly increases the power losses: the filling ratio should be maximized in all studied cases. 

**Figure 6:**
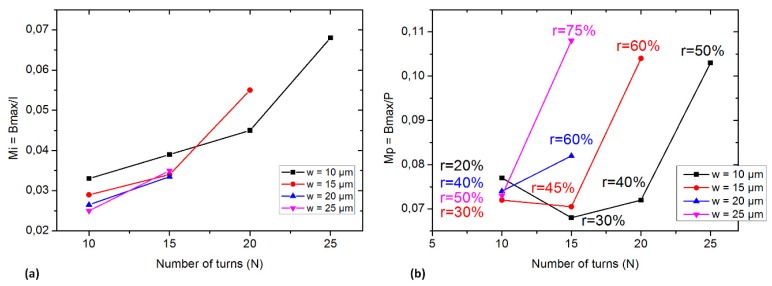
Current and power merit factors M_I_ and M_P_ of coils with R*_ex_* = 500 μm; *s* = 10μm with different turn numbers and wire widths. (**a**). Power merit factor M_I_ is calculated based on the maximum generated magnetic field on each unit of injected current (I) in coils. (**b**). Power merit factor M_P_ is calculated based on the maximum generated magnetic field on each unit of energy consumption. The r ratio represents the filling factor.

A current with low voltage is fed to the trapping thick electroplated copper coils with a power source. The current merit factor M_I_ = *B_max_*/I accounts for the power source efficiency. The power merit factor M_P_ = *B_max_*/P accounts for the amount of B generated per unit Joule heat and can be used to quantify the heating of the coil per unit B generated. Current and power merit factors for different coil configurations are shown in Figure 6.

The M_I_ merit factor curves (Figure 6a) indicate that the optimum coil is obtained when the number of turns of the coils is maximum, i.e., the turns in the center of the coil do not create much B but are a key optimization factor. Furthermore, M_I_ curves suggest using thin wires. Thin wires (10 μm wide) lead to a maximum filling factor of 50%. The magnetic field generated in this case is roughly proportional to N.I, the product of the current per wire multiplied by the number of turns. Thinner wires turning into a lesser value of B_MAX_ is a quasi-straightforward conclusion supported by the results shown in Figure 6a.

In a similar manner, the M_P_ merit factor curves (Figure 6b) indicate that all the configurations of the coils produce a higher B_MAX_ per power loss unit when the inner space of the coil is filled with conductors. Nevertheless, this conclusion is not true if the width of the wires becomes high relatively to the coil’s radius: if the wire’s width increases much, the inner turn cannot be drawn and the inner space must be left empty. The maximum theoretical filling factor then cannot be obtained, hindering the final power merit factor M_P_. Figure 6b shows that for conductors of 10, 15 and 25 μm wide, M_P_ can reach similar values around 0.1. For 20 μm wide coils, the inner turns space is left empty: the inner 4 turns are not designed. M_P_ only gets, then, a value of 0.08, a decrease from 20% to 30%. 

When the inner space is fully filled with conductors, Mp is affected by r: for any coil designed in a way that the coil’s surface is completely filled by conductors, choosing wider conductors makes the filling factor change from 50% for 10 μm wide conductors to 75% in the case of 25 μm wide conductors. The raise in the filling factor enhances M_P_ by approximately 10%.

Finally, the simulations carried out in this paragraph lead us to specify general rules for the design of trapping coils:

- the magnetic field decreases fast with the distance to the coil’s surface: for a given section determined by the liquid flow considerations, the microchannels should preferentially be wide, provided the chosen width does not lead to the microchannels collapse,
- the magnetic field ripple in the coil’s vicinity strongly reinforces the trapping force: the protective layer should be as thin as possible. The protective layer should be much thinner than the conductors’ width,
- the coils conductors should be wide in a manner that the coil surface is mainly supporting the conductors. Empty spaces due to the center connection and the space between turns should be minimized: the windings should fill all the available space.

With these rough design rules, the trapping force can be maximized for a given input power. The heating of the chip is then lowered for a given trapping capability.

## 3. Fabrication of Microfluidic Chip

Integrating planar coils into a bio-chip is quite straightforward. The microfluidic part could be realized as a post-process step. The fabrication process in this study has three main microfabrication steps carried out in a cleanroom: planar coil fabrication, PDMS microchannel molding and microfluidic chip assembly (with thermocouple positioning for temperature measurements) using reversible bonding technique [32].

### 3.1. Microcoil Fabrication

The planar coils parameters were determined previously: square coil (1 × 1; 1.5 × 1.5 and 2 × 2 mm^2^); a range of turns numbers is 15 to 46 turns; the width and height (thickness) of wire are within 15 to 30 μm range and the space between two wires is chosen to a reasonably small dimension of 10 μm, ensuring a 100% fabrication yield in any case (see Table 1). 

**Figure 7:**
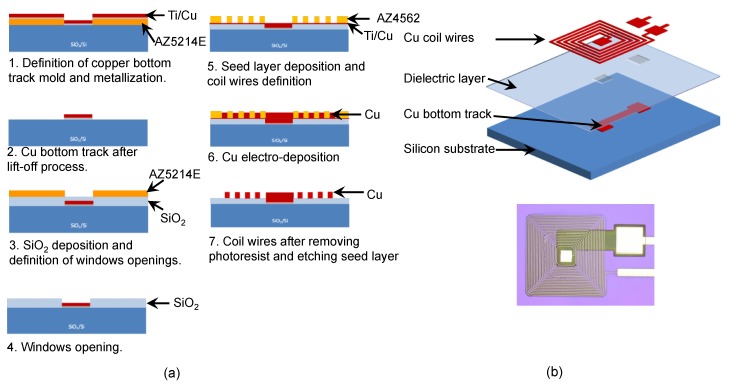
(**a**) Process of microcoil fabrication. (**b**) Exploded CAD view of the microcoil and optical microscope top view (R*_ex_* = 500 μm).

Planar coils are fabricated following the steps (1) to (7), Figure 7a. (1) The first layers of the coil, that consists of a Cu track connecting to the center of the coil, was patterned in 1,5 μm thickAZ5214E photoresist (MicroChem, Westborough, MA, USA) layer by optical lithography (EVG^®^620 Automated Mask Alignment System, St. Florian am Inn Austria). Then, a layer of Ti (10 nm) and Cu (700 nm) was sputtered (EXPLORER Denton Vacuum^®^system, Moorestown, NJ, USA). (2) A lift-off process was performed to obtain the Cu track. (3) The dielectric layer of 500 nm thick SiO_2_ was deposited by PECVD (STS PECVD System, Bristol, UK) using SiH_4_ and N_2_O gases. A 4 μm-thick AZ5214 photoresist was spun over the SiO_2_ layer to pattern the windows to create the contact between bottom Cu track and center wire of the coil. (4) After that, the windows were opened in the dielectric layer by RIE (STS system). (5) A sputtered seed layer of Ti (10 nm)/Cu (100 nm) for coil wire layer was deposited. The mold of coil wires was patterned in a 20 μm thick AZ4562 photoresists (MicroChem, Westborough, MA, USA) by optical lithography. (6) Cu coils were obtained by electrodeposition at the speed of 0.4 μm/min and (7) the seed layer was etched by Argon ion beam (Roth & Rau IonSys 500, Hohenstein-Ernstthal, Germany). Finally, the wafer containing coils was diced in suitable pieces for assembling subsequent microfluidic chip steps.

### 3.2. Microfluidic Chip Fabrication

The microfluidic chip was fabricated in two steps: PDMS channel fabrication and chip assembly. The microcoils could not be put in contact with the liquid flowing in the microchannel. The process detailed here allows the coil protection and further reuse.

#### 3.2.1. PDMS Channel Fabrication 

The channels were realized by PDMS replica molding on a SU8 master: 

(1) The master mold: a 50 μm thick layer of photoresist SU-8 3050 (MicroChem, Westborough, MA, USA) was spin-coated onto a silicon wafer (2 inches). The resist was soft-baked on a hot plate and exposed to UV (with transparent mask, Paris, France), and then post hard-baked. Finally, the developing process is carried out in SU-8 developer solution and the sample is rinsed with iso-propanol. 

(2) PDMS channel: fresh PDMS was prepared by uniformly mixing two components, silicone elastomer-base and curing agent, Sylgard^®^ 184 silicone elastomer kit, Dow Corning Corp., at a ratio 10:1 (wt./wt.). After mixing, the mixture was degassed in a vacuum chamber. Then, the fresh PDMS was poured onto the mold, and cured at 90 °C for 1 h in an oven. 

**Figure 8:**
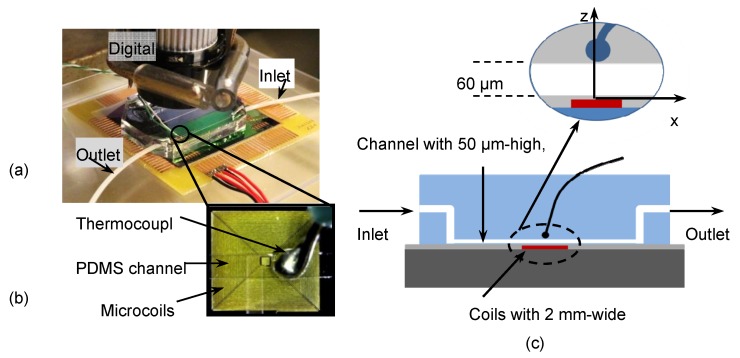
Microfluidic chip with PDMS channel and thermocouple fixed on the top of channel. (**a**). Microfluidic chip with PDMS channel; (**b**). Integrated thermocouple on top of PDMS channel; (**c**). Cut view of the microfluidic channel.

In case the microfluidic chip was used for temperature measurement, a K-type thermocouple was carefully fixed on the top of the channel mold, as close to the channel as possible (Figure 8) before pouring fresh PDMS on. 

Finally, the embossed channel was carefully released from the master mold and cut into a suitable shape. The inlet and outlet of the channel were drilled directly by PDMS tool kits (Figure 8a). The parameters of the channels were typically 50 μm high, 500 μm wide, 3.5 cm long and 1.5 mm diameters for inlet and outlet.

#### 3.2.2. Microfluidic Chip Assembly

CYTOP was used as a low-adhesive layer. The CT-809M (CYTOP, AGC Chemicals Europe, Ltd., Thornton-Cleveleys, UK) 1.5 μm thick layer was spin-coated on the microcoil surface (500 rpm/10 s + 3000 rpm/20 s). Then, this layer was baked on a hotplate at 100 °C for 90 s and annealed in an oven at 180 °C for 1 h. The coils’ substrates were glued on a PCB support and connected by aluminum micro-wire bonding. 

**Figure 9:**
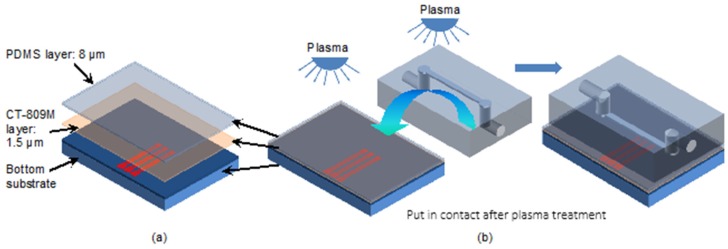
Three coils in line below the microchannel and their connection pads. (**a**) Exploded view of layer structure on microcoil surface; (**b**). Bonding process by using plasma treatment.

Subsequently, the CYTOP coated coil is protected by a PDMS thin layer, which acts as a bonding layer and as a protection layer for electroplated copper. As described in [34] a thin layer of PDMS was spin-coated on the surface (Figure 9a).

The channel cap and the substrate were then oxygen plasma treated for 1 min (oxygen pressure: 0.4 mbar, power: 160 W), put in contact and cured in the oven at 75 °C for 1 h (Figure 9b). 

**Figure 10:**
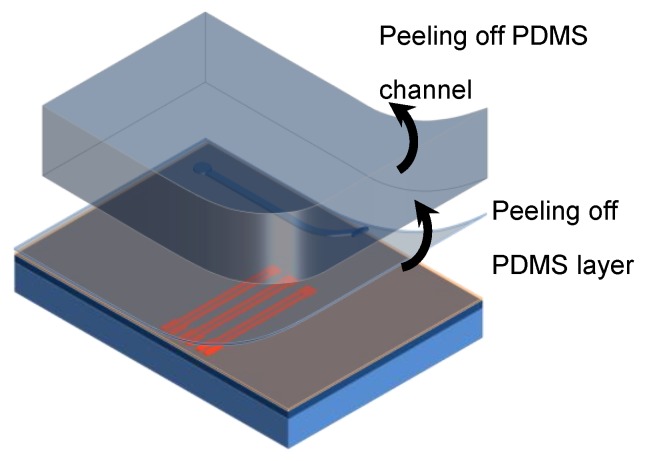
Peeling channel and PDMS layer off bottom substrate.

After bonding, both the channel cap and PDMS coil protective layer can be easily peeled off the bottom substrate (Figure 10). Thus, the bottom substrate can be reused after each experiment carried out in the channel. The reuse process would start by spin-coating a new PDMS thin layer for a new bonding cycle.

These chips are used to test the ability of trapping magnetic nanobeads, and to measure the heat generation of the coils during the trapping experiments.

## 4. Trapping Magnetic Beads Experiments and Temperature Measurements

**Figure 11:**
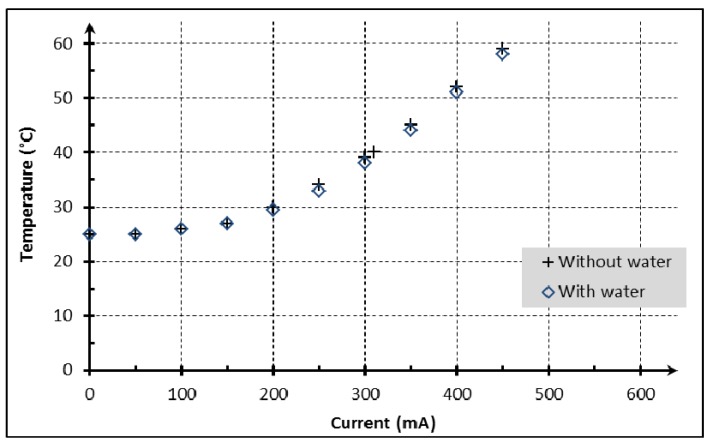
Influence of applied current on temperature in the chip; Embedded coil with parameters: 2 mm wide; *w* = 10 μm; *s* = 10 μm; *N* = 46 turns; *h* = 10 μm.

The experimental setup is represented in Figure 8. A DC current was injected in the coil by a power supply in two conditions: one with the injection of water by pumping, at a continuous flowrate of 12 μL/min, and the other without flow rate through the microchannel. The DC current and temperature were recorded after 10 min to allow thermal stabilization of the chip. Figure 11 shows the measured temperature in the close vicinity of the microchannel as a function of the current flowing through the coil in stationary conditions. 

As shown in Figure 11, the temperature curve as a function of the current in the trapping coil is parabolic. As was expected, the small volume of fluid flowing through the channel compared to the overall volume of the device affects the temperature in a negligible manner. An elevation of temperature at around 60 °C was obtained with a current of 450 mA. This temperature increase is not suitable for microfluidic chips used for antibodies/antigens experiments. 

**Figure 12:**
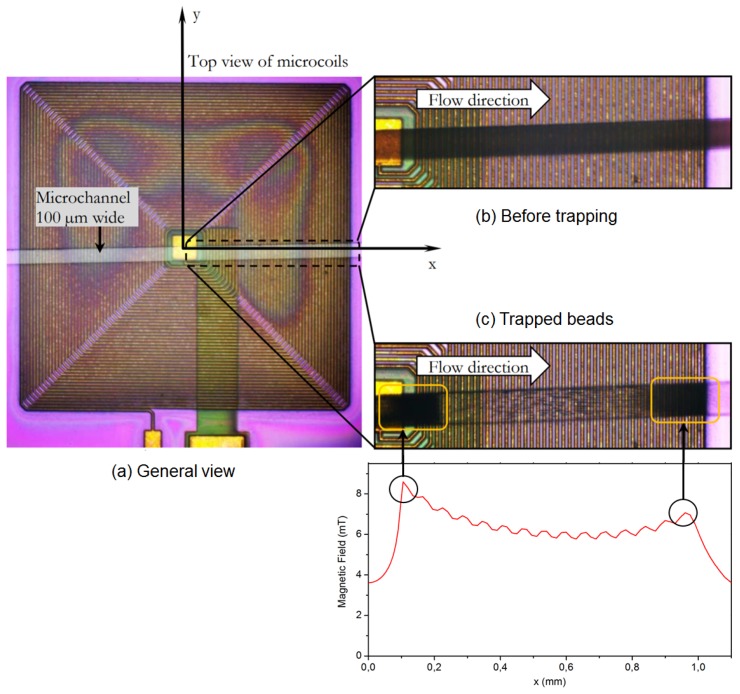
Experiment of trapping beads by coils. Channel parameters: 100 μm wide, 3 cm long and 50 μm high. Coil parameters: 2 mm wide, Cu wire width: 10 μm; space between two wires: 10 μm; Cu wire height: ~15 μm; and 46 turns. Trapping conditions: Beads concentration: ~ 1.33 × 10^11^ beads/100 μL, flow rate: 0.2 μL/min; current I = 120 mA.

The current limit is determined by the biological functions temperature requirements (<41 °C). This temperature is far below the destruction limit of the copper conductors. The trapping of magnetic nano beads was tested in this microfluidic chip with Magnetic Nano-Beads (MNB - Carboxyl-Adembeads, 300 nm, purchased from AdemTECH^®^, Pessac, France), and 120 mA. Some trapping images are shown in Figure 12. 

The beads trapping experiments were performed in a 100 μm wide, 50 μm deep channel (Figure 12a). As showed on Figure 12b,c, beads were trapped with high density at the center and outer edge positions. The simulation results are reminded in the Figure 12. The simulation and experimental trapping results correspond.

The experimental measurements show a concentration of MNB of ~ [2.10^6^ beads/100 μL] +/− [1 × 10^1^ beads/100 μL] on the first peak and ~ [7.10^5^ beads/100 μL] +/− [2 × 10^1^ beads/100 μL] on the second peak, which is consistent with our simulation.

The results of the two experiments demonstrated that our geometry of coils is suitable for effectively trapping magnetic beads in the case of in situ bead-based immunoassay with a negligible temperature raise.

## 5. Immunoassay Complex Validation

In the present paragraph, focus is made on using our microcoils for biological element detection. The aim of this part is to demonstrate the possibility of functionalizing magnetic nano beads (MNB) and using ELISA protocol in order to detect Ovalbumin using microcoils.

### 5.1. ELISA Protocol Description

#### 5.1.1. Beads Grafting

**Figure 13:**
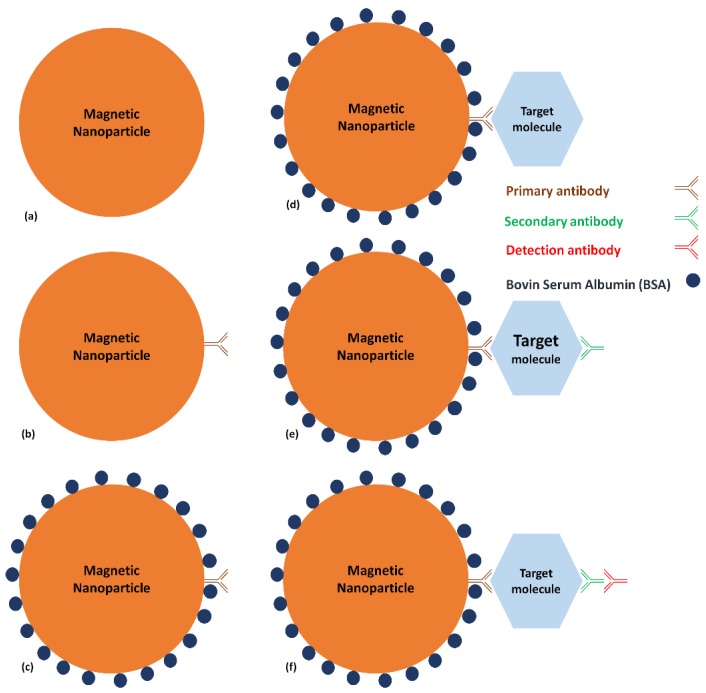
The different steps of an immunoassay with an MNB: (**a**) MNB alone, (**b**), Addition of the primary antibody (**c**) Protection with BSA, (**d**) Addition of the target molecule, (**e**) Addition of the secondary antibody, (**f**) Addition of the detection antibody.

In a test tube, an EDC/NHS protocol, 24 h at 4 °C, was performed to graft primary antibodies on MNB (Figure 13b). In order to prevent non-specific absorption, BSA was added, incubated for two hours at room temperature (Figure 13c). The Ovalbumin was then added (in different concentrations) during two hours at room temperature (Figure 13d). Secondary antibodies were added for two hours at room temperature and finally, detection antibodies were added for two hours at room temperature (Figure 13e,f). After each step a washing step, using a magnet to block MNB, was done to remove all ungrafted biological elements. Finally, the functionalized MNB were observed on glass under the fluorescent microscope.

In a microfluidic system, the ELISA protocol sequence is mainly the same as in macroscopic systems. The single difference is that a step of channel protection is added before the experiment. The total duration of the ELISA process was considerably reduced.

#### 5.1.2. Microchannels Preparation

A HCl/PEO protocol was first used to protect PDMS channels from unwanted antibodies or antigen grafting. The PDMS surface was O_2_ plasma treated before bonding. The microfluidic ports were obstructed to protect the HCl/PEO grafting. The silanol groups at PDMS surface are protonated using a 1M HCl solution (10 μL/min). Then, PEO solution is used for grafting PEO chains on the microchannels walls. The ELISA protocol described at paragraph 0 is carried out after PBS cleaning. The small microchannels volume allows us to speed up the EDC/NHS reaction from 24 h to 30 min at 4 °C regulated temperature. Subsequent steps were carried out in 10 min (2 h at macroscopic scale) at 22 °C. 

In these experiments, the flow rates are determined by magnetic attraction considerations: the biological step requires stronger attraction force than flow forces on the magnetic beads. The biological steps must be realized at lower flow rates. Finally, the ports are closed for the fluorescent microscope observations. 

### 5.2. Validation of Magnetic Immunoassay Using Microcoils

**Figure 14:**
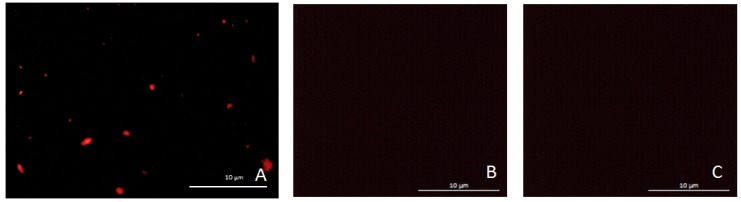
Fluorescent images of (**A**) whole biografting process is respected; (**B**) we skipped the primary antibody grafting step; and (**C**) we skipped the Ovalbumin grafting step. In (**B**,**C**) we showed that immunoassay cannot be performed without this step (we had a great specificity).

Different control experiments were realized to assess the validity of the previously described protocols: an experiment was carried out without the first antibody and another one without the target biomarker (Ovalbumin). These experiments were realized in a tube and in the microfluidic device. Images presented in this paper were extracted from experiments in the microfluidic device. Figure 14a shows a successful immunoassay. Figure 14b,c show the results of control experiments without the first antibody and without the Ovalbumin respectively. 

In conclusion, we demonstrated that by applying magnetic field with microcoils to block MNP and optimize ELISA protocol, we realized successfully an immunoassay. This preliminary result represents a proof-of-concept for using microcoils efficiently in magnetically controlled immunocapture.

**Figure 15:**
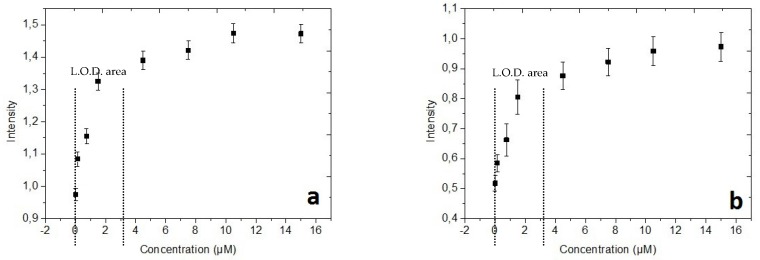
Fluorescent intensity of immunoassay response function of Ovalbumin concentration: (**a**) in microfluidic devices using microcoils; (**b**) in microtubes using a permanent magnet, which allows the LOD calculation from the specific region of linearity of the immunocapture curves.

Moreover, using our controlled MNP trapping system should help us to attain the lowest Ovalbumin concentration that we could detect by optimizing the immunocapture protocol [34]. This information can be reached by performing an immunoassay with different Ovalbumin concentrations. We carried out this experiment and the results are reported in the Figure 15. 

As we expect for an immunoassay response, Figure 15 highlights two features: the first one starts from 0 to 3 μM, which corresponds to the enhancement of the biological complex quantity developed on the surface of MNB. The second one, from 3 to 15 μM, corresponds to a flat zone when all or most bioactive sites have been occupied by a biological complex. In particular, the curve (Figure 15a) of the integrated system for MNB trapping shows a better dynamic of response in the linear zone than the Figure 15b corresponding to the in-microtube standard technique, which proves a quantitatively more sensitive response in the microfluidic system. Additionally, the curve Figure 15a illustrates a higher intensity in the saturation zone (flat zone), which could be interpreted as an enhancement of the occupied bioactive sites in comparison with the in-tube standard technique.

Therefore, to evaluate the sensitivity of the Ovalbumin fluorescence detection using the two strategies of experiment (in microtubes and in microfluidic system), we have calculated the limit of detection (*L.O.D.*) of the target biomarker. The *L.O.D.* was determined using the standard formula commonly used in immunocapture experiments [35,36]:

(8) $$L.O.D.=I_{0}+3*\sigma_{D}$$

where $\sigma_{D}$: standard deviation and $I_{0}$: Intensity with no target (Ovalbumin).

Consequently, the immunocapture curves of the Figure 15 permitted us to determine an L.O.D. of 90 pM for the microfluidic system with the integrated microcoils, far below the in-tube immunoassay which represents an LOD of 2.1 nM. In addition, these reported results corroborate those obtained by our recent complementary work to validate the reliability, in terms of probability of success of the integrated trapping technique to enhance the magnetic immunoassay performances [34].

## 6. Conclusions

The magnetic field generated by planar microcoils was calculated by ANSYS® software at some reference positions. The magnetic field modulus was extracted at these positions and the trapping ability of the microcoils was analyzed from these results. The magnetic field intensity on the distance along the z-axis decreases rapidly. Synthetic merit factors were determined and their dependence on the coils’ parameters was studied. The results show that the planar coils could be designed with the aim of getting the maximum value of B with low energy consumption (high power efficiency).

The width of the wires should be chosen high but much lower than the coils radius. Furthermore, the width and space arrangement should allow the higher filling of the surface.

Considering these results, a set of microcoils was fabricated and integrated in a microfluidic chip. The microfluidic chip was fabricated using an advanced packaging technique for reusing bottom smart substrate and having disposable channels. This reversible assembling of PDMS microfluidic chip overcomes PDMS channels contamination issues in bio-experiments. 

Temperature measurements were carried out in order to evaluate the capabilities of such a chip to effectively trap magnetic nanoparticles. It was found that even if the microcoil is able to generate a magnetic field at its vicinity suitable for nanoparticles trapping, the whole chip suffers a temperature increase that may not be compatible with biological processes. A trade-off between magnetic attraction and temperature management is key for a functional biochip. The chosen condition for the immunoassay was to keep the temperature below 41 °C.

Magnetic immunoassay was successfully realized using the designed microcoils in a microfluidic chip. The immunoassay was carried out successfully in the tube and in the microfluidic conditions: an improvement of the limit of detection (90 pM) was obtained in comparison with 2.1 nM in tube conditions.

## Figures and Tables

**Table 1:** Input parameters and explored values for magnetic field simulation.

Input Parameters for Calculation	Simulated Values
*w* (width of Cu wire)	10, 15, 20, 25, 30, 35, 40, 45, 50 μm
*s* (separation between 2 Cu wires)	10 μm
*h* (height of Cu wire)	15 μm
*N* (number of turns)	10, 15, 20, 25, 30, 35, 40
R*_ex_* (outer radius of the coil)	500, 750 and 1000 μm
*J* (current density)	1.10^9^ A/m^2^ (kept constant)
